# The Effects of High-Fat Diet on the Molecular Pathways in Cardiac Tissue: A Systematic Review of In Vivo Rodent Studies and Integrated Bioinformatic Analysis

**DOI:** 10.3390/biomedicines13092071

**Published:** 2025-08-26

**Authors:** Muhammad Syaffuan Ahmad Najib, Marjanu Hikmah Elias, Norsham Juliana, Siti Hamimah Sheikh Abdul Kadir, Effendi Ibrahim, Nazefah Abdul Hamid

**Affiliations:** 1 School of Pharmacy, KPJ Healthcare University, Persiaran Seriemas, Kota Seriemas, Nilai 71800, Negeri Sembilan, Malaysia; maffuan2005@gmail.com; 2 Faculty of Medicine & Health Sciences, Universiti Sains Islam Malaysia, Persiaran Ilmu, Bandar Baru Nilai 71800, Negeri Sembilan, Malaysia; marjanuhikmah@usim.edu.my (M.H.E.); njuliana@usim.edu.my (N.J.); 3 Faculty of Medicine, Universiti Teknologi MARA, Sungai Buloh Campus, Selangor Branch, Jln Hospital, Sungai Buloh 47000, Selangor, Malaysia; sitih587@uitm.edu.my (S.H.S.A.K.); effendi953@salam.uitm.edu.my (E.I.); 4 Institute of Pathology, Laboratory and Forensic Medicine (I-PPerFoRM), Faculty of Medicine, Universiti Teknologi MARA, Sungai Buloh Campus, Selangor Branch, Jln Hospital, Sungai Buloh 47000, Selangor, Malaysia

**Keywords:** gene expression, gene ontology, high-fat diet, cardiovascular diseases

## Abstract

**Background/Objectives:** The global high prevalence of cardiovascular diseases (CVDs) is attributed to the high prevalence of obesity and metabolic syndrome. However, the impact of a high-fat diet (HFD) on the expression of genes in cardiac tissue remains poorly understood. **Methods:** A thorough literature search was performed using PubMed, Scopus, EBSCOhost, and ScienceDirect databases. The Medical Subject Heading (MeSH) terms such as “high-fat diet”, “gene expression” and “cardiac tissue” were used as the keywords in all fields. **Results:** A total of 1608 studies were retrieved, and only in vivo experimental studies to identify cardiac tissues differentially expressed genes (DEGs) or proteins (DEPs) in rodents fed with HFD were selected. After screening, 14 studies were selected, 159 DEGs and DEPs were extracted from the data and further analysis was conducted employing DAVID, STRING, and Cytoscape 3.10.3 software. A protein–protein interaction (PPI) network revealed a total of 159 genes and proteins from the selected DEGs containing 100 nodes and 292 edges with a PPI enrichment *p*-value of < 1.0 × 10^−16^ and an average local clustering coefficient of 0.447 with an average node degree of 5.84. Six significant clusters with high intermolecular interactions from the protein–protein interaction (PPI) network complex reveal significant molecular pathways, including the glucose metabolic process, fatty acid metabolic process, and inflammatory response (*p* < 0.05). **Conclusions:** The identification of the critical link between obesity and HFD-induced CVDs in cardiac tissue highlights the need for a deeper understanding of the molecular mechanisms controlling gene expression in cardiac tissue.

## 1. Introduction

Cardiovascular diseases (CVDs) remain one of the leading causes of death and disability globally, presenting a significant challenge for public health systems. Among the numerous risk factors associated with the onset and progression of CVDs, diet is one of the most important and modifiable. HFD has garnered increased attention for its correlation with the development and aggravation of cardiac pathologies [1].

A high-fat diet (HFD) results in the accumulation of intracellular lipids and induces insulin resistance. Fat accumulation provokes a massive influx of macrophages and produces pro-inflammatory cytokines, dysregulation of adipokines, insulin resistance, and even atherogenesis [2]. Chronic HFD is associated with an increased risk of stroke and cardiovascular disease. Obesity also may lead to cardiac remodelling induced by a chronic HFD, such as left ventricular hypertrophy and fibrosis. These cardiac changes reduce cardiac reserve and increase the likelihood of myocardial ischemia, myocardial infarction, and cardiac failure, thereby raising patient morbidity and mortality [3]. HFDs alter genes involved in glucose and fatty acid metabolism, increasing Glut1 and Cd36 expression and lowering Cpt1b, while other genes found to be expressed include Ech1, Decr1, Hsd17b4, Hsdl2, and Acadvl, in the cardiac and aortic Co-DEPs that are associated with lipid metabolism and may be helpful as diagnostic and therapeutic targets for obesity-induced cardiovascular disease [4,5]. It was also found that an HFD affects gene expression in heart tissue, with higher Cebpa gene expression in female offspring raised on an HFD [6]. Moreover, myocardial apoptosis is a response to an HFD and suggests a novel function of UCP2 and UCP3 expression [7].

A previous study [8] showed that prenatal exposure to an HFD in animal models is potentially associated with persistent histopathological and molecular changes in the heart before overt obesity onset. In mice, HFD was found to impair lipid metabolism, enhance oxidative stress, and deregulate protein expression associated with fatty acid metabolism in both heart and aorta [5,9]. Functional enrichment of these differentially expressed genes (DEGs) and proteins (DEPs) and pathways, as well as their functions in the cardiac tissue, could help understand effects in the heart. Furthermore, the response of cardiac tissue to HFD involved changes in metabolites, increased fatty acid utilisation, and alterations in antioxidant proteins, indicating adaptations to the dietary intervention at both metabolomic and transcriptomic levels [10].

Transcriptome analysis of obese mice found 184 differently expressed genes, with glucose metabolism playing an essential role in HFD-induced heart remodelling [11]. Nr4a1 has been identified as an essential regulator of glucose metabolism balance in obesity-related cardiovascular disease [11]. Previous studies have demonstrated that functional pathway analysis revealed disruptions in critical signalling cascades in the combination exposure group, including downregulation of the Fgf/Pi3k/Akt pathway and activation of the Pgc1α mitochondrial biogenesis pathway [12]. These findings emphasise the intricate relationship between HFD, cardiac gene expression, and metabolic pathways.

However, the early alterations in gene expression that cause vascular dysfunction, particularly in adolescence, are still poorly understood; only two studies have examined how a short-term HFD affects the expression of specific genes in the aorta [13]. The alterations in genes related to vascular tone regulation suggest that short-term exposure to HFD during adolescence is sufficient to disrupt key biological pathways in the aorta, potentially priming the vascular system for dysfunction [13]. Another study reported that mice fed an HFD during adolescence exhibited clear signs of vascular endothelial dysfunction, suggesting short-term HFD exposure during early life stages is sufficient to compromise endothelial function selectively. This could potentially increase the risk of cardiovascular disease later in life [14].

To date, no comprehensive molecular mechanism of gene expression in cardiac tissue has been established, as many studies are small cohort studies with limited sample sizes. The specific molecular mechanisms through which HFD alters gene expression and contributes to cardiovascular disorders are still not completely understood.

Hence, this systematic review consolidates findings from multiple studies to provide a comprehensive understanding of how HFD affects patterns of gene and protein expression, functions, interactions, and critical pathways in the cardiac tissue of HFD rodents, using integrated bioinformatic analysis.

## 2. Materials and Methods

### 2.1. Research Data

This review was registered in the OSF REGISTRIES (Open Science Framework) (https://osf.io/efg9z) (accessed date 21 August 2025). The PRISMA guidelines were followed when conducting this systematic review. The databases PubMed (https://pubmed.ncbi.nlm.nih.gov/), (accessed date 22 September 2023) Scopus (https://id.elsevier.com/) (accessed date 22 September 2023), EBSCOhost (https://www.ebsco.com/) (accessed date 28 September 2023), and ScienceDirect (https://www.sciencedirect.com/) (accessed date 28 September 2023) were used to conduct an extensive literature search with an unlimited starting publication date until December 2023. Related research papers used the Medical Subject Headings (MeSH) keywords “high-fat diet”, “gene expression”, and “cardiac tissue” as their keywords. MeSH terms from the Cochrane Library were used to create synonyms for the keywords. The following keyword sets were combined (“AND”) as part of the search strategy: (1) “High-fat diet” OR “Dietary fats” OR “High fat diet,” (2) “Gene expression” OR “Gene profile”, (3) “Cardiac tissue” OR “Myocardium” OR “Cardiac muscle” OR “Myocardia”. The bibliographies of the papers that were located contained more references.

Inclusion criteria included in vivo studies reporting gene expression results from qPCR, microarray, or next-generation sequencing (NGS) analysis. Abstracts investigating the DEGs in the cardiac tissue of HFD rodents were included. Protein expressions resulting from immunohistochemistry or Western blot were also included. Only studies that collected rodent cardiac tissues were included to maintain uniformity in sample type and minimise biological variability.

Exclusion criteria included publications of editorials, case reports, narrative reviews, scoping reviews, and systematic reviews. In silico and in vitro studies were also excluded. Studies with no differentially expressed gene list were excluded. Studies that utilised interventions other than HFD were excluded. Moreover, studies that involved diet-induced obese rodents and gene knockout (KO) rodents were also excluded.

### 2.2. Screening Process

**Figure 1:**
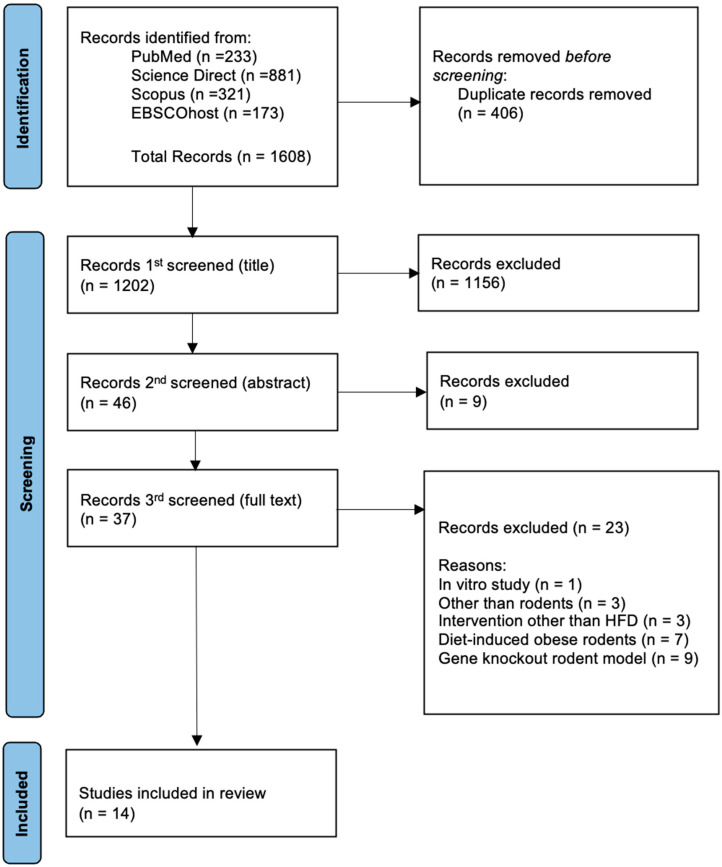
PRISMA flow diagram for study selection in this systematic review that includes the number of records identified from each database, the number of duplicates removed, reasons for exclusions at the full-text stage, and the final number of studies included.

Articles collected from all sources underwent three stages of screening. In the first step, duplicates were eliminated, and all articles with irrelevant titles were disqualified. In the second phase, the abstracts of the remaining papers were reviewed, and those that did not fit the inclusion criteria were eliminated. Lastly, the remaining articles’ full texts were thoroughly reviewed. Systematic reviews, editorials, case reports, narrative reviews, scoping reviews, in vitro, in vivo, and in silico articles, clinical studies, and articles that did not meet the inclusion criteria were excluded in this third phase. All the authors were involved in the screening, selection, and data extraction phase. The PRISMA flow diagram summarises the article selection process and the reasons for article elimination (Figure 1).

### 2.3. Data Analysis Methods

Data were extracted from the studies that fulfilled the inclusion criteria. All the authors participated in extracting the data. A data collection form was used to standardize the data collection, and all data extraction was performed independently. Any disagreements were discussed, and decisions were made based on the majority. Disagreements in study selection were first discussed among three reviewers to reach a consensus. If consensus could not be achieved, the decision was made by involving a fourth independent reviewer. Records on reasons for rejection were kept. The collected data are as follows: (1) author’s name and year, (2) type of rodent, (3) percentage of HFD, (4) duration of feeding, and (5) differentially expressed genes and proteins (Table 1).

#### 2.3.1. Quality Assessment

Three reviewers (MSAN, NAH, and MHE) independently evaluated each paper’s study quality using the Joanna Briggs Institute (JBI) critical appraisal tools. Two additional reviewers (SHSAK and EI) validated the results of the study quality. The studies were graded as low quality (high risk of bias) if the overall score was less than 50%, moderate quality (moderate risk of bias) if the overall score was 50–69%, and high quality (low risk of bias) if the overall score was more than 69%.

#### 2.3.2. Bioinformatics Analysis

All selected DEGs and DEPs from the Venn diagram analysis were pooled and analysed through protein–protein interaction (PPI) functional enrichment analysis using STRING (version 12.0; https://string-db.org/, accessed 15 March 2025) to construct the PPI network [14]. The organism was set to *Rattus norvegicus* with a minimum interaction score of 0.4 (medium confidence), and active interaction sources included textmining, experiments, databases, co-expression, neighbourhood, gene fusion, and co-occurrence. The STRING output was exported into Cytoscape (version 3.10.1; http://www.cytoscape.org/, accessed 15 March 2025) for visualisation of molecular interaction networks and integration of gene expression profiles of DEGs and DEPs [15]. Network module analysis and protein clustering were performed using the Molecular Complex Detection (MCODE) plug-in with the following parameters: degree cut-off = 2, node score cut-off = 0.2, node density cut-off = 0.1, k-core = 2, and max depth = 100. Genes in each identified cluster were subsequently analysed independently using the Database for Annotation, Visualization, and Integrated Discovery (DAVID) (version 6.8; https://david.ncifcrf.gov/tools.jsp, accessed 15 March 2025) with the background set to *Rattus norvegicus*. Gene ontology (GO) terms were considered significantly enriched at a Benjamini–Hochberg adjusted *p*-value < 0.05 [16].

All the genes in each cluster were analysed using DAVID to discover the gene ontology that exhibited significant functional-annotation enrichment related to CVD due to an HFD. The involvement of genes in the CVD pathway was identified based on the cellular components, biological processes, molecular functions, and Kyoto Encyclopaedia of Genes and Genomes (KEGG) pathway.

## 3. Results

### 3.1. Systematic Research

The four databases identified related research publications from 2002 to 2020. A total of 1608 potentially relevant studies were identified using specific keywords (Figure 1). After removing 406 duplicates, the titles of the remaining 1202 papers were screened. During this screening process, 1156 papers were excluded. The abstracts of the remaining 46 papers were then reviewed, which led to a reduction to 37 papers. Following a comprehensive review of the complete texts, 23 papers that did not meet the established inclusion and exclusion criteria were excluded. Ultimately, 14 studies were selected for this systematic review. Homogenized sampling was employed by rigorously adhering to the defined criteria to prevent sampling bias. Table 1 provides a summary of the characteristics of the included studies.

### 3.2. Study Quality

Using JBI critical appraisal tools for systematic reviews, a thorough quality assessment of the included studies was conducted. Supplementary Table S1 includes a detailed quality assessment of the included studies. The included studies comprised twelve high-quality studies, indicating a low risk of bias across key domains such as study design appropriateness, clarity in inclusion criteria, validity of measurement tools and adequacy of statistical analyses. Another two studies received a moderate quality rating, indicating a moderate risk of bias primarily due to limitations in reporting methodology and potential inconsistencies in outcome measurement. No studies were identified as low quality. Overall, the methodological rigor of the included literature was deemed acceptable, thereby supporting the reliability of the synthesized findings.

### 3.3. Identification of DEGs and DEPs in Cardiac Tissue

Following the removal of duplicate entries, 159 DEGs were extracted from all the selected studies. DEPs and overlapping DEGs were reported in a number of studies, including those by Jin et al. (2019) [17], Catar et al. (2015) [21], Jeckel et al. (2014) [22], and Rindler et al. (2013) [24]. These findings were confirmed by both qPCR and Western blot analysis. By verifying transcriptome changes at the level of protein expression, this dual-method approach increased the robustness of the findings.

On the other hand, Han et al. (2018) [18], Jovanovic et al. (2017) [19], and Fujita et al. (2011) [25] found that DEPs were only identified by Western blot without any accompanying transcript-level validation. Additionally, Lima-Leopolda et al. (2013) [23] and Cornall et al. (2011) [26] reported DEGs by qPCR without further protein-level verification. Notably, Marti et al. (2002) [27] was the only study to use semi-quantification by RT-PCR, identifying two DEGs.

### 3.4. Protein–Protein Interaction (PPI) Network and Modular Analysis

**Figure 2:**
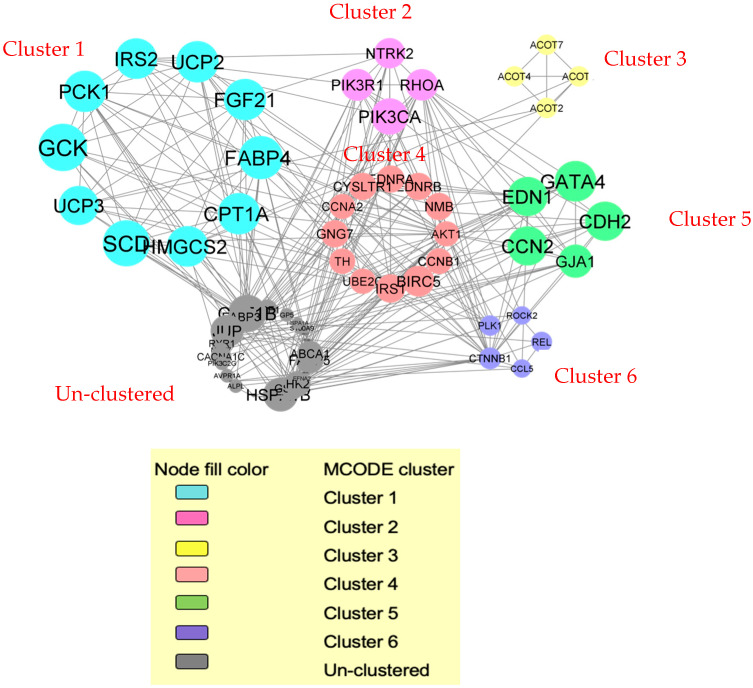
Six significant clusters were created from the PPI network complex generated from the DEGs and DEPs in cardiac tissue.

A total of 159 genes and proteins from the selected DEGs were filtered into a PPI network complex containing 100 nodes and 292 edges with a PPI enrichment *p*-value of <1.0 × 10^−16^ and an average local clustering coefficient of 0.447 with an average node degree of 5.84. The network data was transferred from STRING to the Cytoscape 3.10.3 software to visualize the molecular interaction networks. Using the MCODE algorithm, six significant clusters from the PPI network complex were discovered. Six significant clusters were created from the PPI network complex generated from the DEGs and DEPs in cardiac tissue, in which 6 clusters were identified (Figure 2). The functional-annotation clustering showed that cluster 1 comprises 10 nodes and 34 edges (score = 7.556). While clusters 2 and 3 consist of 4 nodes and 6 edges (score = 4.0), cluster 4 consists of 12 nodes and 21 edges (score = 3.818). Next is cluster 5, which consists of 5 nodes and 7 edges (score = 3.5), and finally cluster 6, with 5 nodes and 6 edges (score = 3.0).

### 3.5. Gene Ontology (GO) and Pathway Enrichment Analysis of the Identified Clusters

KEGG pathway analysis of the DEGs, performed with Benjamini–Hochberg multiple testing correction, identified significant enrichment (adjusted *p* < 0.05) primarily in metabolic pathways and cardiac muscle contraction. Similarly, GO enrichment analysis, corrected for multiple comparisons using the Benjamini–Hochberg false discovery rate (FDR) method, revealed significant enrichment (FDR < 0.05) in biological processes related to cell signalling and energy metabolism. Only pathways and GO terms meeting the significance threshold after FDR correction were reported.

The GO and pathway enrichment analysis showed that the DEGs in cluster 1 were primarily located in mitochondria. These DEGs in cluster 1 involved the biological process of glucose metabolic process (GO:0006006) and regulation of insulin secretions (GO:0050796). Moreover, they are involved in metabolic pathways such as the peroxisome proliferator-activated receptor (PPAR) signalling pathway (hsa03320s) (Figure 2).

The DEGs in cluster 2 were primarily located at the plasma membrane. Their molecular functions are mainly involved in insulin receptor substrate binding, while their biological processes are mainly involved in thrombin signalling and protease-activated receptors. The metabolic pathways of DEGs in cluster 2 include the Ras signalling pathway (hsa04014) and lipid and atherosclerosis (hsa05417).

Meanwhile, the DEGs in cluster 3 were mainly situated in the cytosol. Their molecular function includes long-chain acyl-CoA hydrolase activity (GO:0052816). The DEGs’ biological processes are long-chain fatty acid metabolic processes (GO:0001676), whereas their metabolic pathway is primarily involved in the biosynthesis of unsaturated fatty acids (hsa01040).

The DEGs in cluster 4 were primarily located in the plasma membrane. These DEGs are involved in the cell cycle and mitosis (GO:0051301). Other cardiac-related biological processes include vasoconstriction (GO:0042310) and the regulation of blood pressure (GO:0008217).

Next, the DEGs in cluster 5 were primarily located in the fascia adherens and adherens junction. Their biological processes include positive regulation of I-kappaB kinase/NF-kappaB signalling (GO:0043123) and cell-to-cell signalling (GO:0007267). Their DEGs also involve the arrhythmogenic right ventricular cardiomyopathy metabolic pathway (hsa05412).

Finally, the DEGs in cluster 6 were primarily located in the cytoplasm. The biological processes mainly include I-kappaB kinase/NF-kappaB signalling (GO:0043123) and epithelial-to-mesenchymal transition (GO:0001837). The metabolic pathway for cluster 6 consists of the human cytomegalovirus (CMV) infection (hsa05163). Figure 2 shows the functional annotation clustering of the DEGs with the candidate genes. Table 2 summarises the functional annotation clustering of the DEGS with candidate genes.

## 4. Discussion

Understanding the comprehensive molecular effects of an HFD in cardiac tissues is crucial for developing better screening, management, and treatment of cardiovascular conditions. Thus, numerous investigations on the expression of genes and proteins in heart tissue using diverse techniques have been documented. This systematic review thoroughly investigated gene and protein expression, and their interactions, in cardiac tissue following HFD exposure. From the PPI network and the functional annotation clustering, the discussion is based on how HFD affects gene expression and relevant GO and KEGG pathways in heart tissue. From the 14 selected studies that reported the expressions and interactions in cardiac tissue of HFD rodents, 159 DEGs were detected. The genes and proteins that were highly present in each cluster were selected to compare the level of expression of those in the clusters with the level of genes present in cardiac tissues from other studies.

The KEGG pathway network aims to offer an unbiased viewpoint on possible relationships between enriched pathways. Some genes interact more in a single cluster, as referred to in the GO and pathway enrichment analysis. This analysis is supported by the findings that an HFD altered the expression of nearly 4000 genes, where a small but significant percentage of the differentially methylated regions were found in genes that showed differential expression, indicating a potential link between diet-induced changes and gene expression [27].

The expressed genes in cluster 1 are mainly located in the mitochondrion (GO:0005739). Examples of DEGs include Ucp2, Irs2, Hmgcs2, Pck1, Gck, and *CPT1A*, which are primarily involved in glucose and fatty acid metabolic processes (GO:0006006; GO:0006631). The genes in this cluster interact most with those collected from the selected journals. The expressed genes are involved in the peroxisome proliferator-activated receptor (PPAR) signalling pathway (hsa03320), where their upregulation and fatty acid oxidation pathways are observed in HFD-fed mouse hearts, suggesting lipid excess in the heart may lead to positive coupling of PPAR signalling [28]. In addition, a study found that most genes in the PPAR pathway were significantly overexpressed in HFD as compared to the control diet [29]. This indicates that HFD implies the expressed genes in their molecular functions. Some genes are highly involved in insulin secretion regulation, in which *CPT1A* is associated with the risk of metabolic diseases due to carbohydrate and fat intake [30]. *CPT1A* expression is essential for maintaining whole-body glucose homeostasis, as it supports glucose-stimulated insulin secretion and preserves intracellular ATP levels [31]. Pancreatic expression of *CPT1A* is essential for whole-body glucose homeostasis by supporting glucose-stimulated insulin secretion [31].

Furthermore, Hmgcs2, Ucp3, and Pck1 are increased and expressed in Akita mice hearts, and these highly interacting DEGs correlate with their cardiac expression and the importance of their functions in numerous signalling pathways associated with cardiomyopathy in diabetes [32]. Hmgcs2 overexpression was reported to promote cardiomyocyte apoptosis, inflammation, and oxidative stress, whereas its silencing attenuates these effects in diabetic cardiomyopathy cell models [33]. These findings showed the relationship of the differentially expressed genes with their molecular functions and pathways of glucose metabolism and PPAR signalling pathways in response to HFD effects in heart tissue.

In addition, cluster 2 genes are mainly located at the plasma membrane and include Ntrk2, Pik3ca, Pik3r1, and Rhoa. These genes are primarily involved in pro-inflammatory responses and regulations, as well as the cell differentiation process. Pro-inflammatory responses such as platelet activation, lipid and atherosclerosis pathways, and thrombin signalling and protease-activated receptors indicate an active response to HFD-induced cardiac injury. Thrombin plays a crucial role in driving diet-induced obesity through fibrin-dependent inflammation in adipose tissue [34]. This mechanism demonstrates that obesity leads to chronic activation of the coagulation cascade, significantly increasing the risk of developing metabolic syndrome [35].

The cluster 3 genes mainly consist of the Acot group, which is responsible for the regulation of fatty acid metabolism and controls the oxidation and intracellular trafficking of acyl-CoAs [34]. Located primarily in the cytosol, Acot7, Acot2, Acot1, and Acot4 play a crucial role in the biosynthesis of unsaturated fatty acids and lipid metabolism. It was found that there are significantly altered metabolites and genes, which included the acyl-CoA thioesterase (Acot) family members Acot1, Acot2, and Acot3 (genes of fatty acid metabolic process), particularly Acot2 and Acot3, which increase significantly due to HFDs [36].

The genes in cluster 4 are mainly located at the plasma membrane and in the cytosol. The genes are highly involved in the cell cycle and cell proliferation. The Ednra and Ednrb genes are found to be involved in the regulation of heart rate, blood pressure, and vasoconstriction. The cardiac expression of genes related to the endothelin system, including endothelin receptors A and B, was increased in obese C57BL/6 mice compared to controls [20]. However, it was found that HFD did not affect Endothelin-1 (ET-1) receptor expression (Ednra and Ednrb) in arteries within adipose tissue in adult B6D2F1 mice [25]. These conflicting results may be partly explained as endothelial receptor expression can vary substantially between cardiac tissue and peripheral resistance arteries. Additional factors such as tissue specificity, species or strain differences, developmental stage, methodological sensitivity, and diet duration may further contribute to these inconsistencies. Thus, given that Ednra and Ednrb have substantially promising functions in cardiovascular disease, further investigation is warranted.

Cluster 5 genes are primarily concentrated in fascia adherents. Edn1, Gja1, Gata4, and Ccn2 collectively regulate cell communications in cardiac muscle. Gja1 encodes the protein connexin 43, a gap junction protein essential in cell-to-cell communication and in vascular myoendothelial gap junctions in the heart and other tissues and is downregulated in response to HFD [13]. The expression of Edn1 genes was significantly higher in lean mice compared to obese mice [37], and Gja1 protein expression was reported to remain high in obese adolescent Sprague–Dawley rats, with the same study also noting the transcriptional changes in Gja1 following short-term high-fat diet feeding in adolescent rats [38]. These discrepancies may be explained by species differences (mouse vs. rat), developmental stage (adult vs. adolescent), and tissue specificity, as expression patterns may differ between cardiac tissue, uterine tissue, and vascular beds. Methodological variation, including the use of transcript-level versus protein-level analyses and differences in exposure duration, may also contribute to inconsistent outcomes.

Most cluster 6 genes are expressed in the cytoplasm. Rock2, Ccl5, Plk1, Rel, and Ctnnb1 are mainly associated with inflammatory responses, including positive regulation of IκB kinase/NF-κB signalling and human cytomegalovirus infection. The regulation of fat cell differentiation and negative regulation of I-kappaB kinase/NF-kappaB signalling were the most significantly enriched pathways within D3 (9-amino-acid peptide) treated mice compared with HFD controls [39]. This suggests a correlation between the induction of HFD and the inflammatory response.

For clusters 5 and 6, there was a similar biological process of I-kappaB kinase/NF-kappaB signalling regulations, involving Edn1, Gja1, Rock2, Rel, and Ctnnb1 genes. This could indicate the interaction of these clusters in initiating the inflammatory response of cardiac cells toward the HFD intake. The expression of Edn1 genes in cluster 5 was significantly higher in lean mice compared to the obese [40]. Similarly, Ctnnb1/b-catenin was found to be linked with increased obesity risk in high-fructose and HFD models [41].

A strength of this review is that it includes studies from various rodent species and populations, providing a more comprehensive overview of HFD effects on gene pool expressions. Hence, a more thorough molecular mechanism, biological processes, and molecular pathways of expressed DEGs and DEPs resulting from HFD in cardiac tissue can be identified. Bias reduction and homogeneity of the data were ensured by merely integrating the DEGs and DEPs from qPCR and Western blotting results. The function and role of the genes in the molecular mechanisms, pathways, and biological processes of cardiac tissue resulting from HFD are better understood thanks to the integrated bioinformatic analysis of the DEGs and DEPs compiled from the systematic review.

Nevertheless, the main limitation of this systematic review is the inadequate enumeration of genes that are differentially expressed in some studies and variability in statistical approaches used to determine the DEGs and DEPs. On the other hand, data homogeneity was created by strictly adhering to the inclusion criteria and only choosing DEGs and DEPs identified by qPCR and microarray. To clarify functional downstream pathways of the identified DEGs and DEPs, it is recommended that future research employ targeted gene-silencing techniques, such as knockdown or RNA interference.

## 5. Conclusions

This comprehensive analysis sheds new light on the molecular processes in cardiac tissue associated with HFDs. In addition, the percentage of HFD used in the studies varied significantly, ranging from 20% to 60%. This variability in HFD percentages could have influenced the gene expression profiles and, consequently, the downstream analysis of the DEGs and DEPs.

## Figures and Tables

**Table 1:** Characteristics summary of the included studies.

No	Type of Rodent	HFD (%)	Duration of HFD Feeding	Upregulated Genes	Downregulated Genes	Upregulated Proteins	Downregulated Proteins	Author, Year (References)
1	Mice	60% fat, 20% protein, 20% carbohydrate	8 weeks	93		1	1	Men et al. 2020 [11]
2	Mice	42% kcal fat, 42% kcal carbohydrate	8 weeks	1	-	3	-	Hynynen et al. 2020 [15]
3	Mice	50% saturated, 50% unsaturated fatty acids	8 weeks	2	-	1	1	Plaza et al. 2019 [16]
4	Mice	60% fat	36 weeks	4	3	3	1	Jin et al. 2019 [3]
5	Sprague–Dawley rats	22.5% protein, 45% carbohydrate, 20% fat, 1.3% cholesterol	2 or 4 weeks	-	-	2	2	Han et al. 2018 [17]
6	Wistar female rats	42% fat	10 weeks	-	-	2	4	Jovanovic et al. 2017 [18]
7	Mice	45% fat	11 months	2	-	5	4	Wang et al. 2015 [19]
8	Mice	21% butter fat	10 weeks	4		1		Catar et al. 2015 [20]
9	Wistar rats	57.9% fat	12 weeks	4	3	4	2	Jeckel et al. 2014 [21]
10	Wistar rats	49.2% fat, 28.9% carbohydrates, 21.9% protein	15, 30, or 45 weeks	5	-	-	-	Lima-Leopoldo et al. 2013 [22]
11	Wild type mice (KO)	High fat 60% fat, low fat 10% fat	2,20, or 30 weeks	1	-	1	-	Rindler et al. 2013 [23]
12	Wistar rats	HFD 20% carbohydrates, 60% fat and 20% protein	N/A	-	-	3	-	Fujita et al. 2011 [24]
13	Sprague–Dawley rats	22% fat	12 weeks	1	-	-	-	Cornall et al. 2011 [25]
14	Wistar rats	N/A	65 days	-	2	-	-	Marti et al. 2002 [26]

**Table 2:** Functional annotation clustering of the DEGs with candidate genes.

Cluster	Term	Description	Genes	*p*-Value
1	GO:0005739	Mitochondrion	*CPT1A*, Ucp3, Ucp2, Hmgcs2, Gck	0.002
	GO:0006006	Glucose metabolic process	*CPT1A*, Irs2, Pck1, Gck	5.48 × 10^−6^
	GO:0050796	Regulation of insulin secretion	*CPT1A*, Gck	0.02
	hsa03320	PPAR signalling pathway	*CPT1A*, Fabp4, Scd, Hmgcs2, Pck1	1.78 × 10^−7^
2	GO:0005886	Plasma membrane	Ntrk2, Pik3ca, Pik3r1, Rhoa	0.018
	GO:0016310	Thrombin signalling and protease-activated receptors	Pik3ca, Pik3r1, Rhoa	1.75 × 10^−4^
	hsa05417	Lipid and atherosclerosis	Pik3ca, Pik3r1, Rhoa	0.002
	hsa04014	Ras signalling pathway	Ntrk2, Pik3ca, Pik3r1, Rhoa	2.00 × 10^−6^
3	GO:0005829	Cytosol	Acot7, Acot2, Acot1, Acot4	0.019
	GO:0052816	Long-chain acyl-CoA hydrolase activity	Acot7, Acot2, Acot1, Acot4	1.02 × 10^−9^
	GO:0001676	Long-chain fatty acid metabolic process	Acot2, Acot1, Acot4	8.54 × 10^−6^
	hsa01040	Biosynthesis of unsaturated fatty acids	Acot7, Acot2, Acot1, Acot4	2.70 × 10^−8^
4	GO:0005886	Plasma membrane	Cysltr1, Ednra, Ednrb, Irs1, Ube2c, Gng7, Akt1	0.043
	GO:0051301	Cell division	Ccna2, Ccnb1, Ube2c, Birc5	0.001
	GO:0042310	Vasoconstriction	Ednra, Ednrb	0.012
	GO:0008217	Regulation of blood pressure	Ednra, Ednrb	0.044
5	GO:0005916	Fascia adherens	Gja1, Cdh2	0.002
	GO:0007267	Cell–cell signalling	Edn1, Gja1, Gata4	8.46 × 10^−4^
	GO:0043123	Positive regulation of I-kappa B kinase/NF-kappa signalling	Edn1, Gja1	0.044
	hsa05412	Arrhythmogenic right ventricular cardiomyopathy	Gja1, Cdh2	0.038
6	GO:0005737	Cytoplasm	Rock2, Ccl5, Plk1, Rel, Ctnnb1	0.006
	GO:0043123	Positive regulation of I-kappa B kinase/NF-kappa signalling	Rel, Ctnnb1	7.56 × 10^−5^
	GO:0001837	Epithelial to mesenchymal transition	Rock2, Ctnnb1	0.011
	hsa05163	Human cytomegalovirus infection	Rock2, Ccl5, Ctnnb1	0.004

## Data Availability

All data relevant to the study are included in the article.

## Supplementary Materials

The following information can be downloaded at: https://www.mdpi.com/article/10.3390/biomedicines13092071/s1, Table S1: JBI Critical Appraisal Tools Checklist of Selected Journals.

## Abbreviations

The following abbreviations are used in this manuscript:

CMV	Cytomegalovirus
CVD	Cardiovascular diseases
DAVID	Database for Annotation, Visualization, and Integrated Discovery
DEGs	Differentially expressed genes
DEPs	Differentially expressed proteins
GO	Gene ontology
JBI	Joanna Briggs Institute
KEGG	Kyoto Encyclopaedia of Genes and Genomes
KO	Knockout
MCODE	Molecular Complex Detection
MeSH	Medical Subject Heading
NGS	Next-Generation Sequencing
PPI	Protein–Protein Interaction
qPCR	Quantitative real-time polymerase chain reaction
RT-PCR	Reverse transcription-polymerase chain reaction
STRING	Search Tool for the Retrieval of Interacting Genes/Proteins
