# Supplementary material for: The Effects of High-Fat Diet on the Molecular Pathways in Cardiac Tissue: A Systematic Review of In Vivo Rodent Studies and Integrated Bioinformatic Analysis
Source: Biomedicines. 2025 Aug 26;13(9):2071. doi: 10.3390/biomedicines13092071 (PMC12467030; doi:10.3390/biomedicines13092071)
Supplement: Supplementary file 1 [file biomedicines-13-02071-s001.zip › biomedicines-3814657-SM.pdf]

Supplementary Information

The Effects of High-Fat Diet on the Gene Expression and Pathways in Cardiac Tissue: A Systematic Review of *in vivo* Studies and Integrated Bioinformatic Analysis

Authors: Muhammad Syaffuan Ahmad Najib [a, b], Marjanu Hikmah Elias [a], Norsham Juliana Nordin[a], Siti Hamimah Sheikh Abdul Kadir [c,d], Effendi Ibrahim [c], Nazefah Abdul Hamid [a]

Affiliations:

[a] Faculty of Medicine & Health Sciences, Universiti Sains Islam Malaysia, Persiaran Ilmu, 71800 Bandar Baru Nilai, Negeri Sembilan, Malaysia;

[b] School of Pharmacy, KPJ Healthcare University, Persiaran Seriemas, Kota Seriemas, 71800 Nilai, Negeri Sembilan, Malaysia;

[c] Faculty of Medicine, Universiti Teknologi MARA, Sungai Buloh Campus, Selangor Branch, 47000, Jln Hospital, Sungai Buloh, Selangor, Malaysia;

[d] Institute of Pathology, Laboratory and Forensic Medicine (I-PPerFoRM), Faculty of Medicine, Universiti Teknologi MARA, Sungai Buloh Campus, Selangor Branch, 47000, Jln Hospital, Sungai Buloh, Selangor, Malaysia;

*****Corresponding author email: nazefah@usim.edu.my

Supplementary Table S1: JBI Critical Appraisal Tools Checklist of Selected Journals

No	JBI Checklist Questions	Men *et al.*, 2020	Hynynen *et al.*, 2020	Plaza *et al.*, 2019	Jin *et al.*, 2019	Han *et al.*, 2018	Jovanovic *et al.*, 2017	Wang *et al.*, 2015	Catar *et al.*, 2015	Jeckel *et al.*, 2014	Lima-Leopoldo *et al.*, 2013	Rindler *et al.*, 2013	Fujita *et al.*, 2011	Cornall *et al.*, 2011	Marti *et al.*, 2002
1.	Were the groups comparable other than the presence of disease in cases or the absence of disease in controls?	Yes	Yes	Yes	Yes	Yes	Yes	Yes	Yes	Yes	Yes	Yes	Yes	Yes	Yes
2.	Were cases and controls matched appropriately?	Yes	Yes	Yes	Yes	Yes	Yes	Yes	Yes	Yes	Yes	Yes	Yes	Yes	Yes
3.	Were the same criteria used for identification of cases and controls?	Yes	Yes	Yes	Yes	Yes	Yes	Yes	Yes	Yes	Yes	Yes	Yes	Yes	Yes

4.	Was exposure measured in a standard, valid and reliable way?	Yes	Yes	Yes	Yes	Yes	Yes	Yes	Yes	Yes	Yes	Yes	Yes	Yes	Yes
5.	Was exposure measured in the same way for cases and controls?	Yes	Yes	Yes	Yes	Yes	Yes	Yes	Yes	Yes	Yes	Yes	Yes	Yes	Yes
6.	Were confounding factors identified?	Yes	Yes	Yes	Yes	Yes	Yes	Yes	Yes	Yes	Yes	Yes	Yes	Yes	Yes
7.	Were strategies to deal with confounding factors stated?	Yes	Yes	Yes	Yes	Yes	Yes	Yes	Yes	Yes	Yes	Yes	Yes	Unclear	Unclear
8.	Were outcomes assessed in a standard, valid and reliable way	Yes	Yes	Yes	Yes	Yes	Yes	Yes	Yes	Yes	Yes	Yes	Yes	Yes	Yes

	for cases and controls?														
9.	Was the exposure period of interest long enough to be meaningful?	Yes	Yes	Yes	Yes	Yes	Yes	Yes	Unclear	No	Yes	Yes	Unclear	Yes	Unclear
10.	Was appropriate statistical analysis used?	Yes	Yes	Yes	Yes	Yes	Yes	Yes	Yes	Yes	Yes	Yes	Yes	Yes	Yes
